# The Relationship between Defense Patterns and DSM-5 Maladaptive Personality Domains

**DOI:** 10.3389/fpsyg.2017.01926

**Published:** 2017-11-02

**Authors:** Antonella Granieri, Luana La Marca, Giuseppe Mannino, Serena Giunta, Fanny Guglielmucci, Adriano Schimmenti

**Affiliations:** ^1^Department of Psychology, University of Turin, Turin, Italy; ^2^Faculty of Human and Social Sciences, Kore University of Enna, Enna, Italy; ^3^Department of Law, Libera Università Maria Santissima Assunta (LUMSA), Palermo, Italy

**Keywords:** defense mechanisms, defense patterns, personality, psychopatology, diagnosis

## Abstract

**Aim:** Research has extensively examined the relationship between defense mechanisms (DM) and personality traits. However, no study to date has explored if specific defenses (alone or in combination) are able to predict dysfunctional variants of personality domains, as conceived in the alternative DSM-5 model for personality disorders. This study aimed to investigate the relationship between DMs and DSM-5 maladaptive personality domains among adults.

**Materials and Methods:** Three hundred and twenty-eight adults aged between 18 and 64 years old completed measures on DMs and maladapive personality domains. Regression analyses were performed to determine which DMs predicted the maladaptive personality domains of negative affectivity, detachment, antagonism, disinhibition, and psychoticism.

**Results:** According to psychoanalytic literature, results showed that immature defenses positively predicted maladaptive personality domain scores, whereas mature defenses were generally related with better personality functioning. Moreover, different defense patterns emerged as significant predictors of the maladaptive personality domains comprised in the alternative DSM-5 model for personality disorder.

**Discussion:** Our findings support the view that defense patterns represent core components of personality and its disorders, and suggest that an increased use of immature defenses and a reduced use of mature defenses have a negative impact on the development of personality.

## Introduction

As discussed by Sigmund Freud in early papers (Freud, 1894, 1896), the concept of defense mechanism (DM) was that of a mental operation, usually unconscious, directed against the expression of drives and impulses. The original idea was that DMs serve to control or modulate the expression of unacceptable impulses, to protect the individual from being overwhelmed by the anxiety that would result from conscious recognition of these impulses. This conception was subsequently expanded to include the use of defenses as reactions to external sources of stress as well as to internal forces. Contemporary psychoanalytic authors (Kernberg, 1976, 2005; Kohut, 1977; Bromberg, 1998; Cooper, 1998; Cramer, 2006, 2008; McWilliams, 2011) highlight that DMs have the specific function to protect the self from anxiety, conflict, shame, loss of self-esteem, or other unacceptable feelings and negative thoughts.

Cramer (2008) has conducted a review of empirical studies, which supports these fundamental psychoanalytical assumptions on DMs. Specifically, it emerges from her work that DMs have some operational characteristics. These characteristics can be summarized as follows: DMs may be defined as unconscious mental mechanisms that are directed against both internal drive pressures and external pressures, especially those that threaten self-esteem or the structure and the integration of the self; they develop according to predictable sequences with the maturation of the child; they are part of normal personality functioning; they can lead to psychopathology, if one or more are used excessively; they are distinguishable from one another.

There are different opinions about how many DMs exist (Freud, 1936; Laughlin, 1979; Vaillant and Vaillant, 1992), but there is some agreement between psychoanalytic theorists on the idea that defenses are ordered on a continuum, differing in degree of maturity (Laughlin, 1979; Vaillant et al., 1986; Perry, 1990; Cramer, 1991a; PDM Task Force, 2006; McWilliams, 2011). Generally, DMs that are considered more mature imply a greater ability to adapt to reality, so that they can effectively distance threatening feelings without distorting the reality. Examples of these defenses are sublimation, humor, suppression, altruism. Differently, DMs that are considered immature or even primitive are characterized by severe alteration of painful mental contents and/or radical distortion of external reality. Examples of these DMs are projection, splitting, acting out, and autistic fantasy.

Several researchers have found significant sex differences both in the use of specific DMs and in overall defensive styles adopted by individuals (e.g., Cramer, 1987, 1991a,b, 2006; Vaillant, 1993; Ptacek et al., 1994; Hibbard and Porcerelli, 1998; Mahalik et al., 1998; Watson and Sinha, 1998; Watson, 2002; Petraglia et al., 2009; Furnham, 2012). Research findings showed that women tend to use more internalizing DMs (such as somatization), while men tend to use more externalizing defenses (such as acting out). This is in line with early theoretical and clinical observations suggesting that women find it more difficult to express aggression outwardly and are more likely to turn it against themselves by relying on defenses that modify inner thoughts and feelings. In contrast, men depend more on defenses that locate conflict in the external world, and tend to turn against the object (Freud, 1932; Deutsch, 1944; Erikson, 1964).

Cramer (2006) has observed that psychological health is not only related to mature DMs, but especially to the ability to appropriately use a variety of DMs in different contexts. Several studies showed that a principal or almost exclusive use of immature defense is a risk factor for the development of different forms of psychopathology (Perry and Cooper, 1989; Bloch et al., 1993; Busch et al., 1995; Spinhoven and Kooiman, 1997; Lingiardi et al., 1999; Bond, 2004; Bond and Perry, 2004; Zanarini et al., 2009). In fact, seminal concepts such as the “rigidity” of personality or the “character armor” (Reich, 1933/1979) clearly express the idea that mental health is strictly related to emotional flexibility.

The field of personality assessment is widely informed by studies on DMs (McWilliams, 2011). There is in fact a vast scientific literature that explains the organization and functioning of the personality in the light of defense patterns adopted by individuals. From a psychoanalytic perspective personality styles and organizations are strongly related with specific defense patterns (Kernberg, 1984; PDM Task Force, 2006; McWilliams, 2011). For example, Kernberg (1984) has identified three types of personality organizations that reflect the individual's predominant psychological characteristics and that are based on the individual's identity integration, DMs, and reality testing. The neurotic organization of personality is characterized by identity integration (object constancy), a conserved capacity for reality testing and a prevalent use of mature and neurotic DMs. Borderline personality organization is characterized by a failure in identity integration(identity diffusion), a conserved reality testing when not in condition of distress, and use of immature DMs. The psychotic organization of personality is characterized by lack of ego boundaries, loss of reality testing, and use of immature and primitive DMs.

Psychoanalytic considerations are reflected in basic research on personality. There is general agreement that mature defenses such as humor, altruism, and sublimation are associated with adaptive functioning (Vaillant, 1994, 2000; Bond, 2004) and are related to the presence of favorable aspects of personality. In this context, McCrae and colleagues (McCrae, 1989; McCrae and Costa, 1989; Costa et al., 1991) examined the relationship between DMs and the five-factor model of personality, and they found that the more mature and adaptive DMs were positively correlated with the Big-Five domain of extraversion, openness, and agreeableness, whereas neurotic and immature DMs were positively correlated with higher neuroticism and, to a lesser degree, lower conscientiousness. Likewise, several researches have shown by means of different personality measures and different methods of assessing DMs that an excessive use of immature defenses such as splitting, projection, and denial is related to affective disorders (e.g., depression and anxiety) and to less favorable personality characteristics (e.g., neurotic, borderline, psychotic, dependent, avoidant, narcissistic, and antisocial traits; see Perry and Cooper, 1989; Berman and McCann, 1995; Cramer, 1999, 2003; Lingiardi et al., 1999; Sinha and Watson, 1999; Millon et al., 2004; Bronnec et al., 2005; Bornstein, 2006; Carvalho et al., 2013; Perry et al., 2013).

However, using measures that allow researchers to better compare and discuss findings within a shared diagnostic framework may help clinicians and researchers who are interested in the relationship between DMs and personality features to going beyond the differences among the myriad measures for assessing personality. In this context, the Personality Inventory for DSM-5-Brief Form (PID-5-BF; Krueger et al., 2012) assesses five domains of personality, according to the alternative DSM-5 model for personality disorders American Psychiatric Association, 2013): negative affectivity (which includes personality features such as emotional lability and hostility), detachment (which includes personality features such as intimacy avoidance and suspiciousness), antagonism (which includes personality features such as grandiosity and manipulativeness), disinhibition (which includes personality features such as impulsivity and risk taking), and psychoticism (which includes personality features such as cognitive perceptual dysregulation, unusual beliefs, and experiences). These five domains represent the maladaptive extremes of the five-factor model of personality, which has effectively framed extensive research in the field of personality and psychopathology (Widiger and Costa, 2012).

To the best of our knowledge, no study to date has used the PID-5-BF to explore the relationship between DMs and the maladaptive variants of the five-factor model of personality. In this respect, our research could bridge a gap in literature, and could also highlight potential differences in the role of DMs when maladaptive domains of personality, rather than their corresponding adaptive domains, are considered. Moreover, empirical research on the relationship between DMs and personality factors has usually privileged an approach to the investigation of DMs that was prevalently based on scores on defense styles (i.e., on combination of similar type of defenses, such as suppression, sublimation, humor, and anticipation for indicating mature DMs), whereas it could be also critical to explore how individual DMs are related with different personality domains.

The aim of the present study was to investigate the relationship between DMs and PID-5-BF maladaptive personality domains. In light of previous findings, we hypothesized that gender differences would be observed in DMs, so that males would use more externalizing DMs (such as acting-out), whereas women would use more internalizing DMs (such as somatization). We also hypothesized that an increased use of immature DMs would predict increased scores in maladaptive personality domains, whereas mature DMs would be linked to lower scores in maladaptive personality domains; however, since psychoanalytic literature has consistently linked different types and functions of DMs with different personality styles, we also expected that each maladaptive personality domain would be related to specific DM patterns.

## Matherials and methods

### Participants

The study involved 328 adults (113 males, 34.5%; 215 females, 65.5%). Participants ranged in age from 18 to 64 years old (*M* = 33.40; *SD* = 13.60). The mean years of education was 15.35 (*SD* = 3.55). There were no gender differences in relation to age [*t*_(326)_ = 0.33, *p* = 0.74] or years of education [*t*_(326)_ = −0.18, *p* = 0.86].

### Procedures

After ethical permission by the University IRB for psychological research, participants were recruited in three cities of Sicily (Italy) through public and electronic advertisements (fliers in public places and posts in social network pages). People who contacted the research office were asked to participate in a study on the characteristics of personality. Those who agreed to participate and signed the informed consent were administered two measures for the assessment of DMs and maladaptive personality domains. Participants did not take any compensation for their involvement in the study. At the end of the study, they were debriefed and thanked.

### Measures

The Defense Style Questionnaire-40 (DSQ-40; Andrews et al., 1993) is a self-report instrument including 40 items that measures the individual defensive functioning through 20 DMs (two items representing each of the 20 DMs). The investigated DMs are categorized into the three defense styles: mature (8 items), neurotic (8 items), and immature (24 items). The mature style embraces four DMs: sublimation, humor, anticipation, and suppression. The neurotic style includes undoing, pseudo-altruism, idealization, and reaction formation. The immature style comprises 12 DMs: projection, passive aggression, acting out, isolation, devaluation, autistic fantasy, denial, displacement, dissociation, splitting, rationalization, somatization. An example item is “I often act impulsively when something is bothering me” (related to acting out). In the DSQ-40, participants respond by a nine-point Likert scale extending from one (strongly disagree) to nine (strongly agree). Scores on single DMs are derived by calculating the mean of the two items measuring each defense; scores on defense styles are calculated by averaging the items loading on each style. Thus, scores range from 1 to 9 for each DM and style, and elevated scores point out higher utilization of the target defense/style. The DSQ-40 has been validated in many countries, including Italy (Farma and Cortinovis, 2000). In the present study, reliability coefficients of Cronbach's alpha for mature style, neurotic style, and immature style were, respectively, α = 0.57, α = 0.55, and α = 0.82.

The Personality Inventory for DSM-5-Brief Form-Adult (PID-5-BF; Krueger et al., 2012) is a 25-item self-rated personality trait assessment scale for adults aged 18 and older. The pool of 25 items tap into five different personality domains according to the alternative DSM-5 model for personality disorders (American Psychiatric Association, 2013). Five maladaptive variants of personality-trait domains are evaluated: negative affectivity, detachment, antagonism, disinhibition, and psychoticism; each trait domain includes five items. Each one of them is rated on a four-point scale (from 0 = *very false* or *often false* to 3 = *very true* or *often true*). An example item is “I feel like I act totally on impulse” (related to the domain of disinhibition). The overall measure has a range of scores from 0 to 75, with higher scores indicating greater overall personality dysfunction. Each trait domain ranges in score from 0 to 15, with higher scores suggesting greater dysfunction in the specific personality trait domain. The PID-5-BF has been validated in many countries, including Italy (Fossati et al., 2015). In the present study, Cronbach's alpha for the PID-5-BF total score was 0.86, whereas Cronbach's alpha for the PID-5-BF domains ranged from 0.66 (detachment) to 0.77 (antagonism).

Sociodemographic data were also collected by means of an *ad hoc*-questionnaire.

### Statistical analyses

Statistical analyses included descriptive statistics for all study variables. Two MANOVAs using gender as factor and age and years of education as covariates were performed, to examine the effects of sociodemographic variables on maladaptive personality domains and DMs. Finally, stepwise regression analyses were performed to examine if specific DMs could predict PID-5-BF total score and its domain scores, controlling for sociodemographic variables.

## Results

### Descriptive statistics and group differences

In our sample, the defense patterns mean scores were in the normal range. The same applied for PID-5-BF scores. Descriptive statistics on the measures used in this study are reported in Table 1 for the full sample and differentiated by gender.

**Table 1:** Descriptive statistics of defense mechanisms and maladaptive personality domains.

	**Full sample (*N* = 328) *M* (*SD*)**	**Range (***N*** = 113)**	**Skewness (***N*** = 215)**	**Kurtosis**	**Males *M (SD)***	**Females *M (SD)***
DSQ mature style	4.82 (1.21)	1.13–8.50	−0.007	0.223	4.95 (1.27)	4.75 (1.17)
DSQ neurotic style	4.36 (1.20)	1.25–7.50	0.009	−0.267	4.30 (1.26)	4.39 (1.17)
DSQ immature style	3.72 (1.06)	1.04–6.75	0.289	−0.167	3.71 (1.14)	3.73 (1.02)
Autistic fantasy	3.43 (2.27)	1–9	0.685	−0.594	3.44 (2.13)	3.43 (2.35)
Acting out	4.47 (2.03)	1–9	0.221	−0.673	4.26 (1.91)	4.57 (2.09)
Anticipation	4.90 (1.63)	1–9	0.006	−0.020	4.95 (1.68)	4.87 (1.60)
Denial	2.66 (1.59)	1–9	0.924	0.569	2.72 (1.44)	2.62 (1.66)
Devaluation	3.79 (1.72)	1–9	0.502	0.056	3.68 (1.71)	3.85 (1.74)
Displacement	3.35 (1.84)	1–9	0.497	−0.367	3.28 (2.00)	3.38 (1.75)
Dissociation	3.25 (1.74)	1–9	0.762	0.296	3.45 (1.89)	3.14 (1.65)
Humor	5.59 (1.96)	1–9	−0.359	−0.496	5.82 (1.91)	5.46 (1.97)
Idealization	4.15 (2.00)	1–9	0.211	−0.605	3.93 (1.94)	4.26 (2.00)
Isolation	3.93 (2.17)	1–9	0.268	−0.946	4.07 (2.07)	3.86 (2.23)
Passive aggression	3.85 (1.97)	1–9	0.328	−0.654	3.98 (2.04)	3.78 (1.92)
Projection	2.74 (1.62)	1–9	0.096	0.779	2.86 (1.78)	2.68 (1.54)
Pseudo-altruism	4.27 (1.85)	1–9	0.214	−0.491	4.25 (1.85)	4.28 (1.85)
Rationalization	5.15 (1.68)	1–9	0.036	−0.442	5.20 (1.69)	5.13 (1.67)
Reaction formation	4.69 (1.76)	1–9	0.274	−0.063	4.70 (1.75)	4.69 (1.76)
Somatization	3.79 (2.11)	1–9	0.448	−0.558	3.31 (1.87)	4.05 (2.19)
Splitting	4.25 (2.26)	1–9	0.303	−0.917	4.30 (2.32)	4.22 (2.23)
Sublimation	4.45 (2.08)	1–9	0.160	−0.699	4.34 (1.97)	4.50 (2.13)
Suppression	4.34 (1.87)	1–9	0.150	−0.528	4.72 (1.93)	4.14 (1.81)
Undoing	4.33 (1.77)	1–9	0.048	−0.476	4.35 (1.99)	4.32 (1.64)
PID-5-BF total score	22.00 (10.52)	0–58	0.318	−0.107	22.33 (10.69)	21.83 (10.46)
PID-5 -BF negative affectivity	6.91 (3.31)	0–15	0.005	−0.638	5.96 (3.39)	7.41 (3.16)
PID-5-BF detachment	3.97 (2.84)	0–13	0.631	−0.135	4.19 (2.81)	3.86 (2.85)
PID-5-BF antagonism	2.88 (2.76)	0–14	1.07	1.13	3.56 (3.21)	2.52 (2.42)
PID-5-BF disinhibition	4.41 (2.90)	0–15	0.545	−0.068	4.67 (2.76)	4.28 (2.95)
PID-5-BF psychoticism	3.83 (2.97)	0–13	0.594	−0.279	3.95 (2.62)	3.77 (3.14)

*DSQ-40, Defense Style Questionnaire-40; PID-5-BF, Personality Inventory for DSM-5-Brief Form Adult*.

A MANOVA showed that sociodemographic variables (age, gender, and years of education) are significantly associated with DM scores in the sample [intercept: Wilks' lambda = 0.54, *F*_(5, 320)_ = 13.25, *p* < 0.001, partial η^2^ = 0.47]. However, multivariate tests showed that only age (partial η^2^ = 0.14, *p* = 0.001) had significant main effects in the model. Tests of between-subject effects showed that sublimation (partial η^2^ = 0.04, *p* = 0.006), humor (partial η^2^ = 0.04, *p* = 0.009), suppression (partial η^2^ = 0.03, *p* = 0.029), idealization (partial η^2^ = 0.02, *p* = 0.047), and somatization (partial η^2^ = 0.03, *p* = 0.011) were significantly associated with the factor comprising the sociodemographic variables. The analysis of the main effects of the model variables on the DMs further showed that male gender was linked to higher suppression (partial η^2^ = 0.02, *p* = 0.008), while female gender was linked to higher somatization (partial η^2^ = 0.03, *p* = 0.002); younger age was linked to higher anticipation (partial η^2^ = 0.01, *p* = 0.043), higher autistic fantasy (partial η^2^ = 0.02, *p* = 0.020), and lower sublimation (partial η^2^ = 0.03, *p* = 0.002); higher levels of education were linked with higher humor (partial η^2^ = 0.03, *p* = 0.004) and higher idealization (partial η^2^ = 0.01, *p* = 0.045).

Another MANOVA showed that the effect of sociodemographic variables (age, gender, years of education) on maladaptive personality domains was significant in the sample [intercept: Wilks' lambda = 0.73, *F*_(5, 320)_ = 23.36, *p* < 0.001, partial η^2^ = 0.27]. Multivariate tests showed that gender (partial η^2^ = 0.11, *p* < 0.001), age (partial η^2^ = 0.08, *p* < 0.001), and years of education (partial η^2^ = 0.03, *p* = 0.048) had all significant main effects in the model. Tests of between-subject effects showed that negative affectivity (partial η^2^ = 0.06, *p* < 0.001), antagonism (partial η^2^ = 0.03, *p* = 0.014), disinhibition (partial η^2^ = 0.03, *p* = 0.010), and detachment (partial η^2^ = 0.03, p = 0.024) were significantly associated with sociodemographic variables. In detail, male gender was significantly related with higher antagonism (partial η^2^ = 0.03, *p* = 0.001), while female gender was significantly related with higher negative affectivity (partial η^2^ = 0.04, *p* < 0.001). Higher age was also linked with lowered negative affectivity (partial η^2^ = 0.01, *p* = 0.046) but higher detachment (partial η^2^ = 0.02, *p* = 0.006). Lower education was linked with higher disinibition (partial η^2^ = 0.02, *p* = 0.009).

### Prediction of maladaptive personality scores

A series of six stepwise regression analysis were performed. These included the 20 DSQ-40 defenses mechanisms and the socio-demographics characteristics of participants (gender, age, and years of education) as potential predictors, and the PID-5-BF total and domain scores as dependent variables.

In detail, a first stepwise regression analysis was performed to examine which DMs best predicted PID-5-BF total scores on maladaptive personality (see Table 2). A significant regression equation was found [*F*_(8, 319)_ = 25.00, *p* < 0.001], with *R*^2^ of 0.38. Participants' predicted PID-5-BF total scores is equal to 9.79 (costant) + 0.96 (Acting out) + 1.04 (Autistic fantasy) + 0.64 (Dissociation) − 0.63 (Humor) + 1.34 (Isolation) + 0.79 (Projection) + 0.53 (Splitting) − 0.88 (Suppression). Participant's PID-5-BF total scores increased 0.96 score for greater use of acting out, 1.04 score for greater use of autistic fantasy, 0.64 score for greater use of dissociation, −0.63 score for less use of humor, 1.34 score for greater use of isolation, 0.79 score greater use of projection, 0.53 score for greater use of splitting, and −0.88 score for less use of suppression. These DMs were significant predictors of PID-5-BF total scores on maladaptive personality domains.

**Table 2:** Stepwise regression analyses predicting PID-5-BF Total score.

**Predictors**	***B***	**95.0% Confidence interval for** ***B***		***T***	***p***
		**Lower bound**	**Upper bound**		
Constant	9.79	6.07	13.52	5.17	0.000
Acting out	0.96	0.44	1.47	3.67	0.000
Autistic fantasy	1.04	0.58	1.49	4.52	0.000
Dissociation	0.64	0.05	1.23	2.12	0.035
Humor	−0.63	−1.13	−0.13	−2.48	0.014
Isolation	1.34	0.88	1.79	5.78	0.000
Projection	0.79	0.12	1.45	2.34	0.020
Splitting	0.53	0.06	0.99	2.21	0.028
Suppression	−0.88	−1.42	−0.35	−3.25	0.001

Model: F_(8, 319)_ = 25.00, p < 0.001; Durbin-Watson = 1.77; R^2^ = 0.38

A second stepwise regression analysis was calculated to evaluate which DMs best predicted PID-5-BF negative affectivity domain. A significant regression equation was found [*F*_(10, 317)_ = 14.51, *p* < 0.001], with *R*^2^ of 0.31. Participants' predicted PID-5-BF negative affectivity is equal to 2.01 (costant) + 1.17 (gender), where gender is coded as 1 for male and 2 for female, + 0.30 (Acting out) + 0.22 (Displacement) − 0.23 (Humor) + 0.31 (Isolation) + 0.25 (Projection) + 0.26 (Pseudo-altruism) + 0.25 (Reaction formation), − 0.28 (Suppression) − 0.02 (age in years).

Participant's PID-5-BF negative affectivity domain thus increased 0.30 score for greater use of acting out, 0.22 score for greater use of displacement, −0.23 score for less use of humor, 0.31 score for greater use of isolation, 0.25 score for greater use of projection, 0.26 score for greater use of pseudo-altruism, 0.25 score for greater use of reaction formation, −0.28 score for less use of suppression; moreover, females showed an increased score of 1.17 for negative affectivity, and increase in age corresponded to a decrease of 0.02 in negative affectivity scores. Acting out, displacement, humor, isolation, projection, pseudo-altruism, reaction-formation, suppression, gender, and age were all significant predictors of PID-5-BF negative affectivity domain in the model (see Table 3).

**Table 3:** Stepwise regression analyses predicting PID-5-BF Negative affectivity domain.

**Predictors**	***B***	**95.0% Confidence interval for** ***B***		***T***	***p***
		**Lower bound**	**Upper bound**		
Constant	2.01	0.02	3.99	1.99	0.047
Gender	1.17	0.53	1.82	3.56	0.000
Age	−0.02	−0.05	0.000	−1.99	0.048
Acting out	0.30	0.14	0.46	3.67	0.000
Displacement	0.22	0.04	0.40	2.40	0.017
Humor	−0.23	−0.40	−0.07	−2.77	0.006
Isolation	0.31	0.16	0.47	4.08	0.000
Projection	0.25	0.03	0.46	2.25	0.025
Pseudo-altruism	0.26	0.08	0.43	2.91	0.004
Reaction formation	0.25	0.07	0.44	2.73	0.007
Suppression	−0.28	−0.46	−0.11	−3.20	0.001

*Model F_(10, 317)_ = 14.51, p < 0.001; Durbin-Watson = 1.80; R^2^ = 0.31*.

A third stepwise regression analysis was calculated to explore which DMs best predicted PID-5-BF detachment domain. A significant regression equation was found [*F*_(6, 321)_ = 15.47, *p* < 0.001], with *R*^2^ of 0.22. Participants' predicted PID-5-BF detachment is equal to 1.96 (costant) + 0.04 (age in years) + 0.15 (Autistic fantasy) − 0.20 (Humor) + 0.37 (Isolation) + 0.29 (Projection) − 0.18 (Reaction formation). Participant's PID-5-BF detachment personality domain increased 0.15 score for greater use of autistic fantasy, −0.20 score for less use of humor, 0.37 score for greater use of isolation, 0.29 score for greater use of projection, −0.18 score for less use of reaction formation; moreover, increase in age corresponded to an increase of 0.04 in detachment scores. Autistic fantasy, humor, isolation, projection, reaction formation, and age were all significant predictors of PID-5-BF detachment domain (see Table 4).

**Table 4:** Stepwise regression analyses predicting PID-5-BF Detachment domain.

**Predictors**	***B***	**95.0% Confidence interval for** ***B***		***T***	***p***
		**Lower bound**	**Upper bound**		
Constant	1.96	0.63	3.30	2.90	0.004
Age	0.04	0.01	0.06	3.35	0.001
Autistic fantasy	0.15	0.01	0.28	2.12	0.035
Humor	−0.20	−0.34	−0.05	−2.71	0.007
Isolation	0.37	0.24	0.51	5.43	0.000
Projection	0.29	0.10	0.49	2.93	0.004
Reaction formation	−0.18	−0.34	−0.02	−2.22	0.027

*Model F_(6, 321)_ = 15.47, p < 0.001; Durbin-Watson = 2.02; R^2^ = 0.22*.

Then, a fourth stepwise regression analysis was performed to evaluate which DMs best predicted PID-5-BF antagonism domain. A significant regression equation was found [*F*_(8, 319)_ = 13.13, *p* < 0.001], with *R*^2^ of 0.25. Participants' predicted PID-5-BF antagonism is equal to 1.50 (costant) − 0.94 (gender), where gender is coded as 1 for male and 2 for female, +0.19 (Acting out) +0.28 (Autistic fantasy) +0.22 (Dissociation) − 0.17 (Idealization) +0.20 (Isolation) +0.26 (Pseudo-altruism) − 0.17 (Reaction formation). Participant's PID-5-BF antagonism personality domain increased 0.19 score for greater use of acting out, 0.28 score for greater use of autistic fantasy, 0.22 score for greater use of dissociation, − 0.17 score for less use of idealization, 0.20 score for greater use of isolation, 0.26 score for greater use of pseudo-altruism, − 0.17 score for less use of reaction formation; moreover, males showed an increased score of 1.17 for antagonisms. Acting out, autistic fantasy, dissociation, idealization, isolation, pseudo-altruism, reaction formation, and gender were all significant predictors of PID-5-BF antagonism domain (see Table 5).

**Table 5:** Stepwise regression analyses predicting PID-5-BF Antagonism domain.

**Predictors**	***B***	**95.0% Confidence interval for** ***B***		***T***	***p***
		**Lower bound**	**Upper bound**		
Constant	1.50	0.06	2.93	2.05	0.041
Gender	−0.94	−1.50	−0.38	−3.29	0.001
Acting out	0.19	0.05	0.33	2.71	0.007
Autistic fantasy	0.28	0.15	0.40	4.43	0.000
Dissociation	0.22	0.06	0.38	2.70	0.007
Idealization	−0.17	−0.32	−0.03	−2.35	0.020
Isolation	0.20	0.07	0.33	3.05	0.002
Pseudo-altruism	0.26	0.11	0.42	3.36	0.001
Reaction formation	−0.17	−0.32	−0.01	−2.10	0.037

*Model F_(8, 319)_ = 13.13, p < 0.001; Durbin-Watson = 1.91; R^2^ = 0.25*.

Afterwards, a fifth stepwise regression analysis was calculated to examine which DMs best predicted PID-5-BF disinhibition domain. A significant regression equation was found [*F*_(9, 318)_ = 14.01, *p* < 0.001], with *R*^2^ of 0.28. Participants' predicted PID-5-BF disinhibition is equal to 5.43 (costant) − 0.03 (age in years) − 0.13 (years of education) + 0.42 (Acting out) − 0.44 (Anticipation) + 0.25 (Autistic fantasy) − 0.29 (Devaluation) + 0.25 (Dissociation) + 0.20 (Isolation) + 0.18 (Pseudo-altruism). Participant's PID-5-BF disinhibition personality domain increased 0.42 score for greater use of acting out, −0.44 score for less use of anticipation, 0.25 score for greater use of autistic fantasy, −0.29 score for less use of devaluation, 0.25 score for greater use of dissociation, 0.20 score for greater use of isolation, 0.18 score for greater use of pseudo-altruism; moreover, an increase in age corresponded to a decrease of 0.03 in disinhibition scores, and an increase in education corresponded to a decrease of 0.13 in this domain of personality. All these acting out, autistic fantasy, devaluation, dissociation, isolation, pseudo-altruism, age, and years of education were all significant predictors of PID-5-BF disinhibition domain (see Table 6).

**Table 6:** Stepwise regression analyses predicting PID-5-BF disinhibition domain.

**Predictors**	***B***	**95.0% Confidence interval for** ***B***		***T***	***p***
		**Lower bound**	**Upper bound**		
Constant	5.43	3.49	7.37	5.51	0.000
Age	−0.03	−0.05	−0.01	−2.60	0.010
Years of education	−0.13	−0.22	−0.04	−2.83	0.005
Acting out	−0.42	0.28	0.56	5.76	0.000
Anticipation	−0.44	−0.62	−0.27	−5.03	0.000
Autistic fantasy	0.25	0.12	0.39	3.80	0.000
Devaluation	−0.29	−0.47	−0.11	−3.24	0.001
Dissociation	0.25	0.09	0.42	2.97	0.003
Isolation	0.20	0.07	0.33	2.96	0.003
Pseudo-altruism	0.18	0.03	0.33	2.40	0.017

*Model F_(9, 318)_ = 14.01, p < 0.001; Durbin-Watson = 1.92; R^2^ = 0.28*.

Finally, a stepwise regression analysis was performed to evaluate which DMs best predicted PID-5-BF psychoticism domain. A significant regression equation was found [*F*_(2, 325)_ = 46.316, *p* < 0.001], with *R*^2^ of 0.22. Participants' predicted PID-5-BF psychoticism is equal to 0.93 (costant) + 0.44 (Autistic fantasy) + 0.35 (Isolation). Participant's PID-5-BF psychoticism personality domain increased 0.44 score for greater use of autistic fantasy and 0.35 score for greater use of isolation. Both autistic fantasy and isolation were significant predictors of PID-5-BF psychoticism domain (see Table 7).

**Table 7:** Stepwise regression analyses predicting PID-5-BF Psychoticism domain.

**Predictors**	***B***	**95.0% Confidence interval for** ***B***		***T***	***p***
		**Lower bound**	**Upper bound**		
Constant	0.93	0.25	1.60	2.71	0.007
Autistic fantasy	0.44	0.31	0.57	6.73	0.000
Isolation	0.35	0.22	0.49	5.13	0.000

*Model F_(2, 325)_ = 46.316, p < 0.001; Durbin-Watson = 1.68; R^2^ = 0.22*.

## Discussion

The present study was aimed at exploring the relationship between DMs and maladaptive personality domains. Most of participants in this study reported DM scores in the normal range (Andrews et al., 1993; Farma and Cortinovis, 2000), suggesting a generally adaptive defensive functioning, as it was expected for a nonclinical sample of adults. The more prevalent defenses in the sample were sublimation, humor, anticipation, suppression, reaction formation, and rationalization. Therefore, most participants relied on mature and neurotic defenses. This general pattern of defensive functioning is characterized by generally mature attempts to: (a) reduce a sense of discomfort or unpleasant affections finding ironic elements in difficult situations (as in the case of humor); (b) to turn negative emotions, impulses or thoughts in more positive and socially acceptable behaviors (as in the case of sublimation); (c) to defer an immediate gratification by anticipating and planning the achievement of future goals (as in the case of anticipation); (d) and to control, neutralize, or transform disturbing emotional or cognitive components (such as suppression, rationalization and reaction formation).

We hypothesized to find gender differences in DMs adopted by participants. However, the present findings only partially supported our hypothesis. Despite previous empirical evidence (Cramer, 1987, 1991a,b, 2006; Vaillant, 1993; Hibbard and Porcerelli, 1998) suggests that men and women rely on different DMs, we found that males and females adopted similar DMs to protect themselves from experiencing excessive anxiety and psychological distress. In fact, the MANOVA examining the effect of sociodemographic variables on DM scores did not find a main effect of gender on these scores. However, it should be noted that the analysis of between-subject effects showed a significant gender difference in the use of mature defenses. Males used the defense of suppression significantly more than females, indicating that in our study it was more likely for males to consciously and deliberately pushing down thoughts, desires, urges, and actions that leads to feelings of anxiety, in order to cope with disturbing situations. Moreover, although previous research (Petraglia et al., 2009) has highlighted that males use more externalizing defenses than females, who tend instead to rely on internalizing defenses, our findings showed that women adopted similar externalizing defenses with respect to men. However, females reported greater use of somatization. Somatization allows the individuals to unconsciously avoid painful feelings in their mind and to experience them in form of physical discomfort and symptoms, and it represents an internalizing DM *par excellence* (Freud, 1896). In this DM, the body is the place in which the internal conflict expresses itself, so that it becomes the “theater” of cognitively unprocessed feelings (McDougall, 1989; Granieri, 2011; Barbasio and Granieri, 2013; Granieri and Schimmenti, 2014; Barbasio et al., 2015; Giovannelli et al., 2016). This finding is in line with the previous research that observed somatization as far more common in women (Lipowski, 1988; van Wijk and Kolk, 1997; Hyphantis et al., 2013a,b). As expected within a theoretical framework informed by psychoanalytic research on the use of specific DMs and how they reflect the developmental level of individuals (Vaillant, 1993; McWilliams, 2011), age and, at a lesser degree, years of education, also associated with how the DMs were used among participants.

Also, as expected in a nonclinical sample, the average scores of the PID-5-BF total and domain scores were in the normal range, generally indicating a good overall personality functioning for the majority of the participants (Krueger et al., 2012; Fossati et al., 2013, 2015, 2017; Gervasi et al., 2017). However, we found significant gender differences with respect to the PID-5-BF maladaptive personality domains. Specifically, we found that women showed higher scores on negative affectivity than males. This finding is in line with research using other measures, in which it is commonly observed a higher prevalence of depression, anxiety, and neuroticism among women (Parker and Brotchie, 2010; Van de Velde et al., 2010; Weisberg et al., 2011; Lehmann et al., 2013; Langvik et al., 2016). In contrast, males displayed higher scores on antagonism than females. This result is consistent with many studies, including South et al.'s (2016) study, in which a clear evidence for higher antagonism among males emerged.

Stepwise regression analyses generally provided support for the hypothesis that immature defenses would predict higher scores on maladaptive personality domains. This general finding is perfectly in line with theoretical models, highlighting that defense style can be regarded as an enduring aspect of personality, in which its mature components facilitate good adjustment and mental health, while its neurotic and immature components promote psychopathology (Kernberg, 1967, 1970, 1975, 1984; Vaillant, 1977; McWilliams, 2011). In fact, the regression model predicting PID-5-BF total scores included an increased used of immature (acting out, autistic fantasy, isolation, dissociation, projection, and splitting) as positive predictors, whereas mature defenses (humor and suppression) were negative predictors of PID-5-BF scores in the model. So, immature and mature defenses played an opposite role in our study, with mature defenses reducing maladaptive personality traits, and immature defenses increasing them. Therefore, our finding supports other research in showing that an increased use of immature defenses and a reduced use of mature defenses have a negative impact on the development of personality.

For what concerns negative affectivity, our study showed that this maladaptive personality domain was related to female gender and younger age, but it was also predicted by an increased use of reaction formation, pseudo-altruism, isolation, displacement, projection, and acting out, along with a reduced use of humor and suppression. According to the alternative DSM-5 model for personality disorders, negative affectivity is characterized by personality facets such as anxiousness, emotional lability, hostility, perseveration, lack of restricted affectivity, separation insecurity, and submissiveness (Krueger et al., 2012). Several studies (for a review, see Al-Dajani et al., 2016) had shown positive associations between negative affectivity and avoidant, schizotypal, borderline, and obsessive-compulsive personality disorders. In this sense, it is not surprising that reaction formation, pseudo-altruism, isolation, displacement, and projection were significant predictor of this maladaptive domain of personality. Reaction formation can be often observed in avoidant and obsessive-compulsive personalities (Fairbairn, 1954; Shapiro, 1965; Gabbard and Newman, 2005; McWilliams, 2011) as a pathognomic defense against negative and unacceptable emotions, feelings, thoughts (such as anger, sense of dependence). It allows to deny ambivalence, to control and to transform disturbing emotional or cognitive components into the opposite polarity to make it less threatening. So, it can be hypothesized that an increased use of reaction formation was related to the tendency of participants with increased negative affectivity to deny feelings of dependence and to try showing greater autonomy; moreover, they may strive to acquire full control over anger by showing them obedient and obsequious. In this context, pseudo-altruism was another significant and positive predictor of negative affectivity. An intriguing hypothesis could be that displaying apparently altruistic and prosocial attitudes might represents an attempt to obtain approval and support from others in people displaying high levels of negative affectivity. Isolation may add to this picture, as it allows individual to manage anxiety and other painful mental states by separating the affective features of an experience or idea from its cognitive dimension. Furthermore, displacement and projection of impulses, emotions, worries, behaviors, and inner aspects of the self may help people high in negative affectivity to defend themselves from overwhelming feelings that are unconsciounsly perceived as potentially disorganizing. In this context as discussed by several scholars (e.g., Kernberg, 1967, 1975; Andrulonis, 1991; Gunderson, 2001), acting out represents a central DM in borderline personality, indicating a tendency to an immediate discharge of feelings or impulses for the inability to endure them and to reflect on the painful circumstances that determined them. In addition, the reduced use of humor and conscious suppression may further reinforce the presence of negative feelings and the difficulty to deal with them.

Regression analyses further showed that older age, higher levels of autistic fantasy, isolation, and projection and lower levels of humor and reaction formation predicted detachment scores. The personality domain of detachment seems to be associated with avoidant, obsessive-compulsive, and schizotypal personality disorders (see Al-Dajani et al., 2016), and it expresses personality facets such as anhedonia, depressivity, intimacy avoidance, suspiciousness, withdrawal. In this sense, it is not surprising that autistic fantasy was a significant predictor of this maladaptive domain of personality. Psychoanalytic literature (e.g., Guntrip, 1952, 1961; Fairbairn, 1954; Eigen, 1973) suggests that autistic fantasy represents the pathognomic defense of the schizoid personality organization against a conflict between the desire to get in touch with others and the fear of being engulfed and overwhelmed by others, which often leads these individuals to social withdrawal. Thus, autistic fantasy may help people high in detachment to defend themselves from other people who could be perceived as overly controlling and invading. In this context, the reduced use of reaction formation could indicate a lower tendency of these individuals to deny ambivalence. The role of isolation in predicting detachment among participants is in line with the psychoanalytic literature, which suggests that isolation is among the organizing defenses of obsessive-compulsive individuals (Fenichel, 1928). It is recognized that these people overestimate mental and cognitive activity and avoid emotionally charged situations for fear of losing control and being vulnerable (Shapiro, 1965). Also, projection emerged as significant predictor of detachment. Projection concerns a wrongly attribution of one's own unrecognized impulses, feelings and thoughts to others, so that the individual can avoid to deal with internal experiences that could make him or her too feel excessively vulnerable. From a psychodynamic point of view, it is possible to hypothesize that increased scores on isolation and projection may help people high in detachment to defend themselves from unconscious feelings of shame and inadequacy. These feelings are likely related to their substantial lack of relatedness (Schimmenti and Caretti, 2016), and the reduced use of humor might further increase, in a vicious circle, the intensity of such unconscious feelings.

Together with male gender, a combination of higher levels of isolation, dissociation, autistic fantasy, pseudo-altruism, and acting out, and lower levels of idealization and reaction formation predicted antagonism scores. Antagonism includes personality facets such as attention seeking, callousness, deceitfulness, grandiosity, manipulativeness (Krueger et al., 2012), and it was found to be associated with antisocial and narcissistic personality disorders (Al-Dajani et al., 2016). According to Kernberg (1975), a grandiose self is present in these personalities, which can be conceptualized as a defense against investment in others and dependence on others. The “pseudo-self-sufficiency” may allow individuals with increased antagonism traits to deny any need for care and love, and to defensively exclude from consciousness disturbing feelings such as anger and resentment toward a needed but frustrating or rejecting figure, which might explain the role of isolation and dissociation in predicting the antagonism domain scores. Autistic fantasy also resulted as a significant predictor in the regression model, suggesting the general tendency of individuals with antagonistic traits to do not engage emotionally with other people. Also, acting out emerged as a significant predictor in the regression model, indicating the tendency of people with high antagonism to act in potentially aggressive and destructive ways toward others and even themselves. These considerations might also explain the increased use of pseudo-altruism in people with higher scores on antagonism: an intriguing hypothesis could be that some people high in antagonism show a facade of altruistic and prosocial attitude, as they are apparently oriented toward others' well-being; however, it is possible that this facade hides a very different attitude, so that desires for power are enacted by unconsciously forcing other people to feel submissive and obliged toward the antagonistic individual. In line with this interpretation, it is not surprising that people high in antagonism display decreased use of idealization. This highlights their lower investment in values and capabilities of others. In addition, the reduced use of reaction formation could indicate a lower tendency of these individuals to feel guilt and to repair potential damage that they inflicted to others.

Regression analyses showed that younger age and lower levels of education predicted disinhibition scores. This domain includes specific personality facets such as distractibility, impulsivity, irresponsibility, lack of rigid perfectionism, and risk taking (Krueger et al., 2012) and it was found to be associated with antisocial and borderline personality disorders (Al-Dajani et al., 2016). The findings on the role of socio-demographic variables in antagonism are consistent with empirical research and clinical interpretations (e.g., Arnsten and Casey, 2011; Charnigo et al., 2013; Klimstra et al., 2014) suggesting that a tendency to act impulsively is related to younger age, when the pre-frontal cortex is not totally mature and behavioral and mental states are not fully integrated into a consistent sense of self, and to lower education that reduce the possibility to use symbols and words to resolve the internal conflicts (Schimmenti, 2016). Along with the role of sociodemographic variables, higher levels of acting out, dissociation, isolation, autistic fantasy, and pseudo-altruism, and lower levels of devaluation and anticipation, were found as significant predictors of disinhibition scores in the sample. The predictors in this model fit very well with Kernberg's (1975) early descriptions of narcissistic, borderline, and antisocial personalities. Specifically, acting out is considered one of the most representative modalities of the borderline functioning. So, it is plausible that people with high levels of disinhibition may act impulsively due to their difficulty integrating internal representations, reflecting on experience, and verbalizing feelings when they face stress and disturbing emotions (Clarkin et al., 2007). Indeed, Freud (1901, 1914) had earlier specified that acting out was a repetitive impulsive behavior acted upon by the individual for a difficulty in communicating. Also, dissociation emerged as significant predictor, suggesting the possible lack of the sense of continuity in the areas of identity, memory, conscience, or perception among participants with high disinhibition. This defensive mechanism may allow to keep the illusion of psychological control when in front of overwhelming mental states, such as hopelessness, hatred feelings, and loss of control. In this respect, psychoanalytic literature (e.g., Bromberg, 1998; Schimmenti, 2016; Schimmenti and Caretti, 2016) suggests that dissociation may paradoxically protect the individuals from a fragmentation of the self through multiple disconnections in the self. Likewise, isolation resulted as a significant predictor in our sample, consistent with the tendency of people with high disinhibition to exclude painful feelings (such as anxiety, shame, guilt) associated with specific events or memories from consciousness. Moreover, autistic fantasy resulted as a significant predictor of disinhibition scores. This might result from the tendency of people with high disinhibition to do not engage emotionally with others, preferring to focus on their personal wishes and needs, and to act on the basis of such internal experiences. Moreover, surprisingly and somewhat counterintuitively, higher levels of pseudo-altruism and lower levels of devaluation added to the predictive model of disinhibition. Increased use of pseudo-altruism could suggest that altruistic behaviors in people high in disinhibition may serve to generate intense, albeit volatile, relationships that may help these individuals to satisfy personal needs. Likewise, a reduced use of devaluation could indicate a tendency to value others as they can support the disinhibited individual and provide help to him or her when needed. Finally, it is not surprising that the reduced use of anticipation was found as a significant predictor in the predictive model of antagonism. People who are impulsive, irresponsible, and prone to risky behaviors usually display considerable inability to delay gratification and to anticipate and plan the achievement of future goals (e.g., Whiteside and Lynam, 2001).

Higher levels of isolation and autistic fantasy predicted psychoticism scores. This domain includes personality facets such as eccentricity, cognitive perceptual dysregulation, unusual beliefs and experiences (Krueger et al., 2012), and it was found to be associated with borderline and schizotypal personality disorders (Al-Dajani et al., 2016). This pattern of defense may reflect the difficulty of people who report high psychoticism to effectively deal with emotional conflicts (Schimmenti et al., 2017), which might lead them to compartmentalize the emotional aspects of the experience. This condition may imply a tendency to exclude the painful feelings from their correspondent cognitive representations (as expressed by increased isolation), and to be absorbed in excessive fantasies as an unconscious strategy to avoid such painful feelings (as expressed by increased autistic fantasy).

In conclusion, our study supports the general hypothesis that maladaptive personality traits are modulated by dominant defense patterns. However, as with every research our study comes with a number of limitations. The study involved only adult volunteers from an Italian isle, which strongly limits the generalizability of our results. Studies on community-dwelling individuals from different cultures and studies with clinical samples are greatly needed to develop this line of research, and efforts directed toward longitudinal research could also offer valuable contribution to better understand the role of DMs in normal and abnormal personality. Also, although the measures used in this study have displayed adequate psychometric properties in worldwide research, it is to be acknowledged that we collected the information by means of self-report measures, which are susceptible of social desirability and cultural bias.

However, our cross-sectional findings support the psychoanalytic consideration that a correct identification of defense patterns is critical for personality assessment, and may be particularly informative for psychotherapy work with individuals who display high levels in one or more maladaptive personality domains.

## Author contributions

AS designed the study. LL, SG, and GM were responsible for data collection. SG, GM, and FG conducted literature searches, and provided summaries of previous studies. LL, FG, and AS conducted the statistical analysis. LL and AG wrote the first draft of the article. AS and AG contributed with relevant theoretical and clinical inputs and were responsible for subsequent collation of inputs and redrafting. All authors critically revised the manuscript and approved its final version.

### Conflict of interest statement

The authors declare that the research was conducted in the absence of any commercial or financial relationships that could be construed as a potential conflict of interest.
